# Analyte Sensing with Catalytic Micromotors

**DOI:** 10.3390/bios13010045

**Published:** 2022-12-28

**Authors:** Mihail N. Popescu, Szilveszter Gáspár

**Affiliations:** 1 Física Teórica, Universidad de Sevilla, Apdo. 1065, E-41080 Sevilla, Spain; 2 International Centre of Biodynamics, 1B Intrarea Portocalelor, 060101 Bucharest, Romania

**Keywords:** catalytic micromotor, self-propulsion, motion-based sensing, enhanced diffusion coefficient

## Abstract

Catalytic micromotors can be used to detect molecules of interest in several ways. The straightforward approach is to use such motors as sensors of their “fuel” (i.e., of the species consumed for self-propulsion). Another way is in the detection of species which are not fuel but still modulate the catalytic processes facilitating self-propulsion. Both of these require analysis of the motion of the micromotors because the speed (or the diffusion coefficient) of the micromotors is the analytical signal. Alternatively, catalytic micromotors can be used as the means to enhance mass transport, and thus increase the probability of specific recognition events in the sample. This latter approach is based on “classic” (e.g., electrochemical) analytical signals and does not require an analysis of the motion of the micromotors. Together with a discussion of the current limitations faced by sensing concepts based on the speed (or diffusion coefficient) of catalytic micromotors, we review the findings of the studies devoted to the analytical performances of catalytic micromotor sensors. We conclude that the qualitative (rather than quantitative) analysis of small samples, in resource poor environments, is the most promising niche for the catalytic micromotors in analytical chemistry.

## 1. Introduction

**Figure 1:**
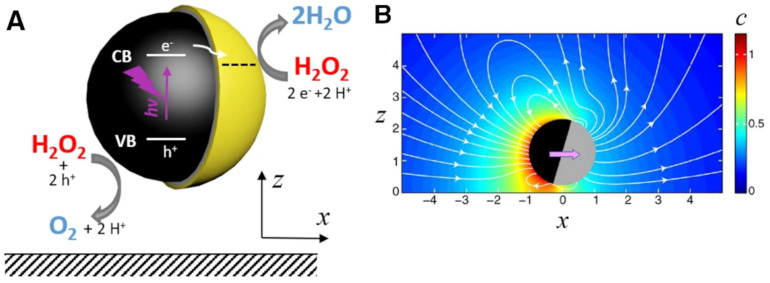
(**A**) Schematic representation of a Janus-type catalytic micromotor self-propelling by the photochemical decomposition of H_2_O_2_ in the vicinity of a wall; (**B**) Calculated concentration gradients produced by a Janus-type catalytic micromotor in the vicinity of a wall (color coded, in arbitrary units) superimposed over calculated hydrodynamic flows produced by such a catalytic micromotor (streamlines, white); Figures reproduced with permission from Ref. [14].

Catalytic micromotors are micrometer-sized objects which self-propel when the solution in which they are suspended contains species which they can chemically convert. It is important to note that in order to achieve self-propulsion, the object should be, in general, sufficiently asymmetric either in shape or/and in the surface distribution of the catalytic processes (e.g., only on half of a spherical microbead, see Figure 1) such that a preferred direction can be defined. (The breaking of the isotropic symmetry, which is necessary for motion, can also be induced by boundaries; e.g., a spherical catalytic particle near a wall would exhibit motion in the direction normal to the wall.) While at short times the motion of a typical micromotor is ballistic, at long times it crosses over to Brownian motion, albeit with an effectively enhanced diffusion coefficient that depends on the activity of the particle [1].

The first catalytic micromotors were reported in 2004: bimetallic nanorods (1–2 µm long, ~200 nm in diameter, half Au and half Pt), which self-propelled by catalyzing the decomposition of H_2_O_2_ [2]. This report has been followed by intense research efforts aimed at, among others: determining the mechanism of propulsion [3,4], the development of micromotors propelling with other “fuel” than H_2_O_2_ [5,6,7], controlling the trajectory of the micromotors [8,9,10], and, obviously, related to the question of applications for catalytic micromotors [11,12]. The efforts aimed at determining the mechanism of propulsion, for example, have shown that self-propulsion can occur via self-diffusiophoresis [1], self-electrophoresis [3,4], or bubble ejection [13].

**Figure 2:**
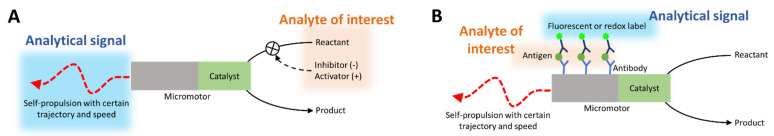
Two ways of using catalytic micromotors for sensing. (**A**) Fuel molecules (or species which inhibit/activate the catalytic process) are the analytes of interest and either the speed or the diffusion coefficient of the catalytic micromotors is the analytical signal; (**B**) The self-propulsion of the catalytic micromotors has the role of increasing mass transport within the investigated sample (by inducing flow of the solution and by moving through the solution). The catalytic micromotors carry classic biomolecule detection schemes (such as the “sandwich-type” detection scheme based on antibodies schematically shown here). Depending on the detection scheme, in this approach, the analytical signal is either optical or electrochemical.

One very promising application of catalytic micromotors is in analytical chemistry, in the detection and quantification of different molecules of interest in food, environmental, or clinical samples. Catalytic micromotors can be used to detect molecules of interest in several ways (see also Figure 2). The most straightforward way is to use the micromotors to detect their “fuel” (i.e., the species they are consuming for self-propulsion) (see Figure 2A). This approach requires the motion of the micromotors to be carefully analyzed (using a microscope, a digital camera, and the appropriate software tools) because the speed (or the diffusion coefficient) of the micromotors, which depends on the concentration of the analyte of interest, is the analytical signal. Another possibility is to use the micromotors to detect species which do not act as fuel for the micromotor but still can modulate (e.g., inhibit, activate, etc.) the catalytic processes facilitating self-propulsion. This approach also requires an analysis of the motion of the micromotors because the speed (or diffusion coefficient) of the micromotors remains the useful analytical signal. Finally, catalytic micromotors can also be used as tools for enhancing the mass transport (and the probability of specific recognition events) in the sample. This approach is based on “classic” (e.g., electrochemical, fluorescent, etc.) analytical signals and does not require an in-depth analysis of the motion of the micromotors (see Figure 2B).

Catalytic micromotors are often obtained by complicated, multistep procedures. Many of them also rely on rather expensive materials (e.g., noble metals, purified enzymes, antibodies, etc.). Cost-effective mass production of highly reproducible catalytic micromotors is still a problem in spite of improvements brought about by, for example, Pickering emulsion-based methods [15,16]. Why are catalytic micromotors still interesting for sensing in such conditions? There are several features which recommend catalytic micromotors for sensing:

(i). First, catalytic micromotors can analyze tiny samples. They can be suspended and their motion investigated in few microliters of sample. This is useful when large volumes of samples are not available (e.g., blood samples collected from very low birth weight infants [17,18,19]). One should also note that the ability to work with small samples advantageously translates into smaller amounts of chemical/biological waste.
(ii). Second, catalytic micromotors (via their motion) can facilitate enhanced mass transport within the investigated sample without using laboratory equipment (such as stirrers, vortexes, or pumps). This can be important for investigations carried out outside specialized laboratories, in remote areas with limited resources. It can also be important when analyzing a few microliters of sample (e.g., a drop of blood, sweat, tear, or saliva placed on a microscope glass slide). There are very few tools for stirring/mixing within tiny liquid droplets.
(iii). Third, the signal of the catalytic micromotors can be documented using a mobile phone instead of a bulky, expensive laboratory equipment. In turn, this can facilitate sensing outside specialized laboratories and, eventually, by untrained users. While this possibility represents an advantage over classic analytical approaches (which most often require bulky and expensive instrumentation that is used by trained staff in specialized laboratories), only a few times it has been demonstrated. Both the collective behavior of catalytic micromotors [20] and the individual behavior of catalytic micromotors [21,22,23] were already documented using mobile phone cameras and linked to the concentration of the analyte of interest. Reading the fluorescence of catalytic micromotors using a mobile phone was also recently reported [24]. Important to note, some of these mobile phone-based approaches to study micromotors are still relying on image analysis carried out on a computer.
(iv). Forth, sensing with catalytic micromotors is characterized by high spatial resolution because each tiny motor reports on the concentration of the analyte in the solution adjacent to the micromotor. However, achieving sensing with high spatial resolution (i.e., building high resolution chemical 2D/3D maps with catalytic micromotors) is currently still hindered by the heterogeneity of the catalytic micromotors. One cannot be 100% sure that a micromotor self-propels faster/slower than the other because of the local availability/unavailability of the targeted analyte or because of intrinsic, but yet not well understood, heterogeneity from batch to batch, or even within the same batch, in the properties of the individual micromotors (see also Section 2), or for various other reasons related to the experimental setup. For example, it has been observed that hydrazine-fueled micromotors propel faster at the edges of a water droplet than in the middle of it, immediately after the droplet is exposed to hydrazine vapors [25]; this is so because in that setup the hydrazine vapors reach the micromotors faster through the shallow edges of the water droplet [25].
(v). Last but not least, catalytic micromotors can combine sensing with other functions (e.g., with neutralization of dangerous chemicals). However, this possibility was also seldom explored. Metal ions were both detected and collected/removed using some H_2_O_2_-propeled catalytic micromotors [26,27].

The above advantages (or possible advantages) of catalytic micromotors as sensors are currently explored by research groups worldwide and were highlighted in a good number of papers. The present (focused and thorough) overview of the catalytic micromotors used to detect and quantify analytes of interest complements several recent reviews about sensing with micromotors [28,29,30,31,32,33,34,35,36,37].

## 2. The Speed (or Diffusion Coefficient) of Catalytic Micromotors as Analytical Signal

When considering potential ways to employ catalytic micromotors as sensors, it is intuitively appealing to attempt exploiting the dependence of the speed (or, alternatively, the effective diffusion coefficient) of the micromotor on the presence of the analyte of interest. This dependency can occur in several scenarios. The analyte of interest can be the reactant in the catalytic process responsible for the self-propulsion, that is, the analyte of interest acts as fuel for the catalytic micromotors. For example, micrometer sized objects modified with enzyme were used as sensors for the substrate of the respective enzyme [6,38]. The analyte of interest can be an inhibitor or activator of the catalytic process responsible for self-propulsion. For example, Au-Pt bimetallic nanorods were used to detect Ag^+^ ions as these ions activated the decomposition of H_2_O_2_ self -propelling such nanorods [39]. Finally, in certain situations, the analyte of interest can slow down the micromotor despite limited (or no) impact on the catalytic process responsible for self-propulsion. For example, the already mentioned Au-Pt nanorods were observed to be slowed down by common ions in rather low concentrations because ions impact the reaction-induced self-generated electric field [39,40].

**Figure 3:**
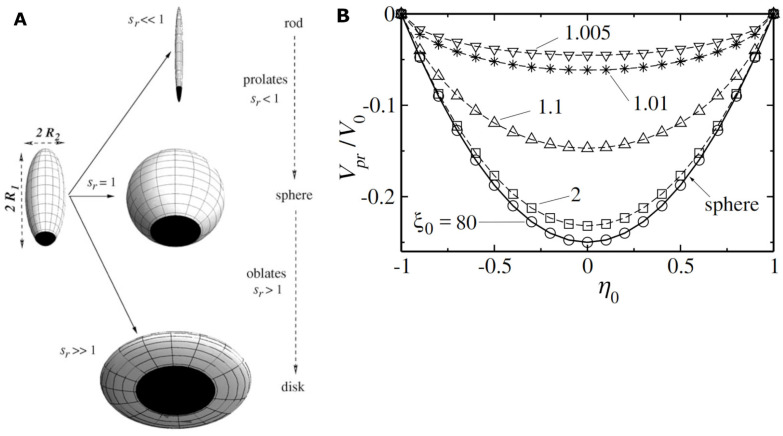
(**A**) Schematic representation of spheroidal catalytic micromotors characterized by the same polar semi-axis (*R*_1_), different equatorial semi-axes (*R*_2_), and, thus, different aspect ratios *s_r_* = *R*_2_/*R*_1_. (Obs.: The part of the catalytic micromotor covered by catalyst, that is, the active cap of the catalytic micromotor, is depicted as a black area). (**B**) The dependence of the scaled phoretic velocity (*V_pr_*/*V*_0_) on the fraction of the surface of the catalytic micromotor covered by the catalyst (*η*_0_ = −1, 0, 1, respectively, correspond to a micromotor surface with no catalyst, with the lower half covered by the catalyst, and completely covered by the catalyst, respectively), for a prolate-shaped spheroidal catalytic micromotor with aspect ratio parameter *ξ*_0_ = (1 − *s_r_*^2^)^−1/2^ = 80 (*s_r_* = 0.9999), 2 (*s_r_* = 0.866), 1.1 (*s_r_* = 0.42), 1.01 (*s_r_* = 0.14), and 1.005 (*s_r_* = 0.099). The large *ξ*_0_ values correspond to a quasi- spherical shape, while the limit towards 1 corresponds to needle-like shapes (approximating a long rod). Figures reproduced with permission from Ref. [41].

In the following, we focus, for simplicity, on the case of self-phoretic spheroidal catalytic micromotors (see Figure 3A), with the analyte of interest being the reactant in the first-order chemical reaction powering the micromotor.

One notes that from a theoretical perspective, this set-up seems optimal, in that the speed is maximal at half-coverage of the micromotor with catalyst (see Figure 3B). The speed is also quite robust against fabrication defects, if the axial-symmetry is preserved: the speed maximum is relatively flat with respect to the coverage, and thus variations in coverage have small influence on the value of the speed [41]. Furthermore, in the range of few micrometers of sizes, and for first-order kinetics of the catalytic chemical reaction, the self-phoretic speeds are independent of the size of the particle [1].

Using a spherical shape as an example, the arguments above can be made more quantitative in that the speed *V*, as a function of the coverage *θ* (the angle between the axis of the particle and the rim of the active cap; it runs from 0, for zero coverage, to *π*, for a fully covered sphere) by the catalyst, is given by *V* = (1 – *cos*^2^*θ*) *V*_0_ (where *V*_0_ ~ *C* is a characteristic velocity directly proportional with the concentration *C* of fuel and, in a first-order approximation, independent on the size of the particle). Thus, in order to see, for example, a change in velocity by 10% from the nominal value at *θ* = *π*/2, which is the case of a Janus particle, the coverage *θ’* should change to the one obeying *cos*^2^*θ*’ = 0.1, i.e., *θ’ ≈* 0.4 *π*. This is a rather large variation, and most of the modern methods of manufacturing Janus colloids can perform better than that without particular technical demands.

**Figure 4:**
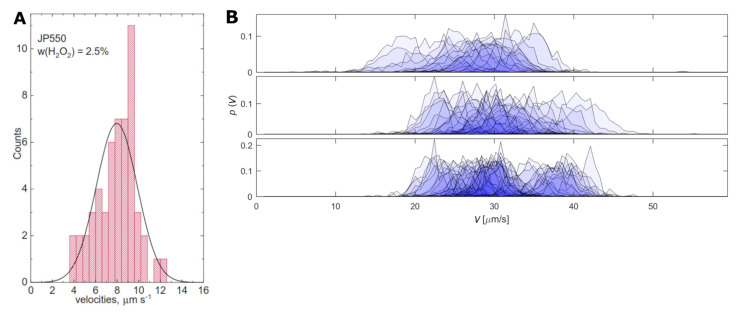
(**A**) Velocity distributions for TiO_2_ on SiO_2_ Janus-type motors (d ~550 nm) in 2.5% H_2_O_2_; (**B**) Velocity distribution for Pt on polystyrene Janus-type motors (d ~2.8 µm) in 0.1% (top), 1.0% (center) and 3.0% (bottom) H_2_O_2_; (Obs.: (**A**) is built using the average speed of individual motors while (**B**) shows experimental velocity distributions for ~30 individual micromotors). Figures reproduced with permission from Ref. [42] (panel (**A**)) and Ref. [43] (panel (**B**)).

Although the theoretical point of view suggests a robust operation of Janus particles as motors, there is an increasing body of experimental evidence that there must exist additional parameters, beyond the geometrical aspects discussed above, that play an important role in the emerging motion. For example, for seemingly identical TiO_2_ on SiO_2_ Janus-type motors (with radius of 275 ± 8 nm), the active velocity is measured by Sachs et al. [42] to be a stochastic variable with a quasi-Gaussian distribution of a width comparable to the average value (7.9 µm/s; see Figure 4A).

Similar findings are reported for nanorods (either classic Au-Pt or the more rapidly moving ones made with carbon nanotubes) [44], as well as for a variety of other types of Janus spheres in aqueous solutions of H_2_O_2_ [43,45] (see Figure 4B). In all cases, this has been attributed to an intrinsic variability of the catalytic activity of the materials, but why this is happening, and how can it be controlled, remains thus far unclear. The impact of such large dispersion around the average value as in the data in Figure 4A is dramatic in what concerns the accuracy of an analytic method based on the value of the velocity (which, in the first approximation, is proportional to the fuel concentration): a change in the velocity from the average value of ~8 µm/s by a standard deviation of ~2 µm/s represents a 25% variation which, unknowingly, would render an over (or under) estimated fuel concentration by the same percentage.

The experimentally observed variability of the speed of catalytic micromotors impacts also the way catalytic micromotors are used as sensors. It is clear that the speed of a large sample of micromotors must be averaged into an average speed both during the calibration of the micromotors with solutions containing known concentrations of analyte and during the analysis of samples of unknown chemical composition. Only such average speeds will reflect the correct analyte concentration in the standard solutions and investigated samples. The wider the distribution of self-propulsion speeds, the larger the number of micromotors which must be analyzed for correct results. Analyzing the speed of large numbers of micromotors requires time, careful experiments (e.g., the experiments must have an optimal number of micromotors in the field of view, the micromotors must stay long enough in the field of view, etc.), and also important computing power. The experimentally observed variability of the speed also compromises the idea of using catalytic micromotors for sensing with spatial resolution (an idea that is based on the assumption that all catalytic micromotors taken into work are characterized by similar parameters of the motion in similar experimental conditions).

In spite of the above-described complication, the first studies exploring catalytic micromotors as sensors did use the speed (or the diffusion coefficient) of the micromotors as the analytical signal (see, for example, [39]). Therefore, the following two sections will shortly review catalytic micromotor sensors which report on the concentration of the analyte of interest via the parameters of their motion. As we will see, the analyte of interest is either the fuel of the catalytic micromotors (Section 3) or a molecule which modulates the catalytic process without being the fuel of the catalytic micromotors (Section 4).

## 3. Sensing Analytes Which Are Also Fuel for the Catalytic Micromotors

Most catalytic micromotors developed up to now use H_2_O_2_ as fuel. These H_2_O_2_-fueled micromotors have been reported to exhibit dependence of the velocity on the concentration of H_2_O_2_. For example, already the first catalytic micromotors, developed by Paxton et al., self-propelled at speeds of 3.9 µm/s in 0.03% H_2_O_2_ and at speeds of 7.9 µm/s in 3.3% H_2_O_2_ [2]. Ni on Pt microtubes (L ~10–20 µm, ϕ ~2–3 µm) were shown to self-propel at speeds of 75 µm/s in 1% H_2_O_2_ and at speeds of 165 µm/s in 5% H_2_O_2_ [46]. Polycaprolactone microspheres (d ~30–40 µm) carrying MnO_2_ particles were also shown to self-propel at speeds of 15 µm/s in 5% H_2_O_2_ and at speeds of 43 µm/s in 25% H_2_O_2_ [47]. As a consequence of this repeatedly documented dependence, the idea of using catalytic micromotors as sensors for the quantification of their fuel molecules has emerged. However, a convincing practical implementation of the idea was slowed down by several findings. Particles presumably moving by self-electrophoresis or self-diffusiophoresis (two mechanisms of self-propulsion) were found to be very sensitive to the presence of ions in the solution, which is actually a ubiquitous feature in most real-world samples [39,48]. The speed of catalytic micromotors was found to disadvantageously depend on the sample matrix as well. For example, Mg-based catalytic micromotors (which self-propel by ejecting H_2_ bubbles in H_2_O, a mechanism considered more robust than self-phoresis) were found to self-propel with speeds of 296 ± 40 µm/s in water, 223 ± 38 µm/s in whiskey, 108 ± 18 µm/s in milk, and only 40 ± 8 µm/s in serum at the same H_2_O_2_ concentration [49]. The speed of catalytic micromotors was found to depend significantly also on the working temperature. For example, the speed of Pt-based rolled-up microtubes (self-propelling due to the O_2_ bubbles produced in 1% H_2_O_2_) was found to be ~400 µm/s at 20 °C and ~550 µm/s at 25 °C [50]. These additional dependencies (of the self-propulsion speed on sample features which are not always easy to control) very much weaken the robustness and reliability of catalytic micromotors as analytical tools. They add to the already mentioned problem related to the variability of the speed of self-propulsion for seemingly similar catalytic micromotors (Section 2).

The few studies in which catalytic micromotors are used as sensors for the detection of their fuel are shortly presented in Table 1. As highlighted in the table, the catalytic micromotors used to sense their fuel self-propel by either self-diffusiophoresis or self-electrophoresis. The fascinating details of these two mechanisms are nicely presented in a review by Moran and Posner [40].

**Figure 5:**
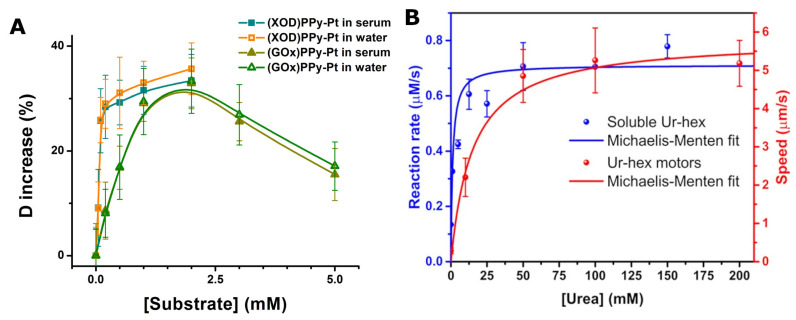
(**A**) The dependence of the relative diffusion coefficient of XOD-modified nanorods and of GOx-modified nanorods on the concentration of hypoxanthine and glucose, respectively, in water and in 10× diluted serum; (**B**) The dependence of the reaction rate (in blue) and of the self-propulsion speed (in red) of silica microcapsules modified with urease on the concentration of urea in water; Figures reproduced with permission from Ref. [6] (panel (**A**)) and from Ref. [38] (panel (**B**)).

Figure 5 completes Table 1 and shows typical calibration curves which link the speed (or the diffusion coefficient) of the micromotors to the concentration of the fuel (i.e., analyte of interest). On one hand, the rather similar calibration curves obtained in water and serum (see Figure 5A) argue that enzyme-based micromotors can be used for sensing in relatively complex samples, such as 10× diluted serum. On the other hand, these calibration curves also highlight yet another difficulty in using enzyme-based micromotors to detect their fuel molecules: the dependence of the speed (or the diffusion coefficient) of the micromotors on the concentration of the fuel is linear for a very narrow range of concentrations. (Moreover, in the case of GOX-based micromotors, there is the additional complication of the re-entrant behavior, i.e., for a range of increases of the diffusion coefficient, there are two possible values of the substrate concentration for each specific value of that increase.) The samples to be investigated must be diluted or preconcentrated in order to fit that range. However, while not convenient, this is not something unusual with other analytical tools as well.

## 4. Sensing Analytes Which Modulate the Speed of Self-Propulsion without Being Fuel for the Catalytic Micromotors

**Figure 6:**
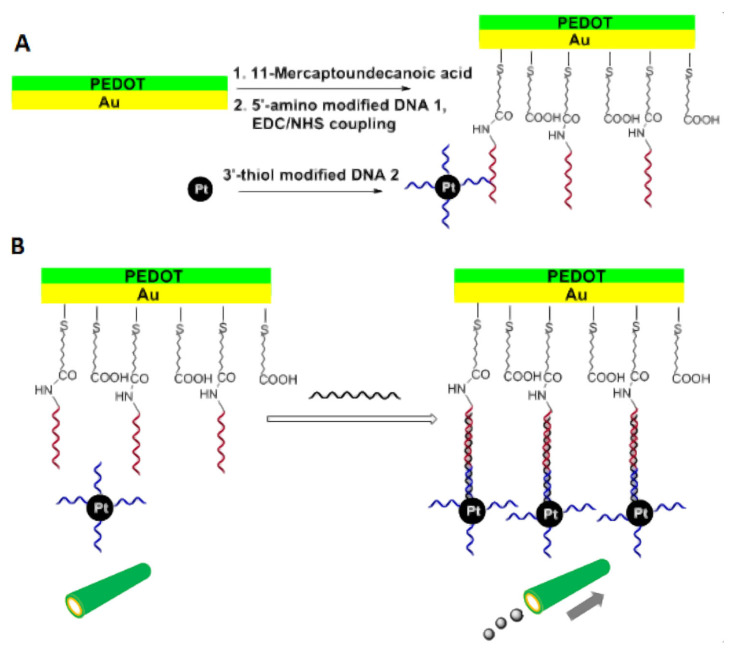
(**A**) Modification of the inner surface of poly(3,4-ethylenedioxythiophene) (PEDOT) on Au microtubes with capture DNA, and of Pt nanoparticles with detector DNA; (**B**) The principle for DNA detection by introducing Pt nanoparticle-DNA conjugates into the microtubes via specific DNA hybridization mediated by target DNA; Figure reproduced with permission from Ref. [51].

As noted in the previous section, catalytic micromotors were used only a few times to detect analytes which also act as fuel for them. We have discussed a number of complications which seem to explain the limited popularity of that approach. However, catalytic processes can be slowed down or accelerated by certain species other than their reactants, and the catalytic processes which self-propel the catalytic micromotors make no exception from this rule. This opens the possibility to use catalytic micromotors also for the detection of these inhibitors and activators. As another possibility, catalytic activity (instead of being slowed down by inhibitors or accelerated by activators) can be conferred to microstructures (e.g., a microtube) from scratch, via biorecognition events selective for the analyte of interest. This approach was used to detect DNA, for example (see details in [51]). The detection required microtubes to be modified with capture DNA. When the target DNA was available in the investigated sample, the modified microtubes were able to bind not only target DNA but also detector DNA that was previously labeled with Pt nanoparticles. The latter, together with H_2_O_2_ as fuel, put the microtubes into motion with speeds which were proportional with the concentration of DNA target in the sample (see Figure 6). Catalytic micromotors facilitated not only this “signal on” (or OFF-ON) type biosensing concept but also “signal off” (or ON–OFF) type detection concepts (see, for example, in Ref. [52]). As a final possibility, one can exploit the fact that accumulation of antibody–antigen–antibody complexes on the surface of catalytic micromotors can slow down the self-propulsion of catalytic micromotors by attaching significant weight to the catalytic micromotors [53].

A summary of the studies employing catalytic micromotors to detect species which modulate the speed of the self-propulsion without being fuel for the catalytic micromotors is presented in Table 2.

There are several interesting points one can make based on Table 2. Most catalytic micromotors listed in Table 2 self-propel by the O_2_ bubbles generated by the decomposition of H_2_O_2_ and not the phoretic mechanisms mentioned in Section 3. This mechanism of self-propulsion was most probably preferred due to its relative insensitivity to common ions. Bubble-propelled catalytic micromotors were already tailored for the detection of a wide range of analytes, such as small metal ions (e.g., Hg^2+^), nucleic acids (e.g., DNA), proteins (e.g., carcinoembryonic antigen), viruses (e.g., Zika virus), and small organic compounds (e.g., glutathione). Most catalytic micromotors listed in Table 2 use the speed of self-propulsion as the analytical signal. However, this speed can be replaced with the distance traveled by the catalytic micromotors as the analytical signal. Such a switch, from speed to distance, was already done for the detection of DNA [59].

On the other hand, the drawbacks and problems of the catalytic micromotor sensors mentioned in Section 3 are carried over also to the catalytic micromotor sensors listed in Table 2. Using the speed of catalytic micromotors as the analytical signal still requires highly reproducible micromotors (as slightly different motors will report slightly different concentrations of the targeted analyte) and obtaining highly reproducible micromotors is still very difficult. For example, the average speed of some catalytic micromotors used to detect DNA in the nM range was reported to be 418 ± 25 μm/s [52], while such a standard deviation corresponds to tens of nM of DNA. pH sensitive catalytic micromotors were observed to self-propel at speeds in between 70 µm/s and 110 µm/s at the same pH of the solution [57]. Such a difference in the speed of self-propulsion (40 µm/s) corresponds to roughly 2 pH units. Averaging the speeds of several catalytic micromotors is clearly needed in order to correctly determine the concentration of the targeted analyte in a sample. As already pointed out, averaging is time consuming (as several trajectories need to be analyzed) and compromises the possibility of using catalytic micromotors for biosensing with high spatial resolution. Micromotors listed in Table 2 are still significantly slowed down in complex media, such as cell culture media, serum, or whole blood. For example, poly(aniline) on Pt microtubes were observed to self-propel with speeds of 140 μm/s in buffer solution with 1.2% H_2_O_2_ and with speeds of 90 μm/s in serum with 1.2% H_2_O_2_ [53]. The slowdown, when operating in serum, was speculatively attributed to the higher viscosity of the medium as compared to the buffer solution. A rather strong dependence of the speed of bubble-propelled catalytic micromotors on the temperature was also re-confirmed by the study in Ref. [58]. Although it was not mentioned in Section 3, the duration of the self-propulsion at a constant speed could also become a problem when the speed of the catalytic micromotors is used as the analytical signal. For example, catalytic micromotors built with catalase were reported to have a constant speed only for about 2–3 min [52,58]. Such a short time-period characterized by self-propulsion at a constant speed might be a problem in the hands of untrained users (who might read the speed of the catalytic micromotors at too long times, and, thus, draw incorrect conclusions regarding the concentration of the analyte of interest). Catalytic micromotors characterized by a constant self-propulsion speed for long time periods (e.g., 5–10 min) are needed when such structures are to be used for reliable biosensing.

## 5. Sensing Analytes Which Are Not Involved at All in the Catalytic Process Propelling the Micromotors

Section 3 and Section 4 describe the way catalytic micromotors can be used to sense analytes which impact the catalytic process propelling the micromotors, and, thus, the motion of the micromotors (see also Figure 2A). However, catalytic micromotors can also be built to detect analytes which are not involved at all in these catalytic processes (see also Figure 2B). In such cases, the catalytic micromotors are used to enhance the analyte-proportional analytical signals in several ways:

(i). By enhancing mass transport, and, thus, enhancing the probability of the biorecognition event to happen. Enhancing mass transport by the self-propulsion of catalytic micromotors comes with some advantages. For example, it does not require laboratory equipment (e.g., magnetic stirrers, shakers, vortex mixers, etc.), and, thus, it is suitable to be used both in specialized laboratories and outside specialized laboratories, in resource poor areas. Enhancing mass transport by the self-propulsion of catalytic micromotors can also be expected to eliminate some previously described inconsistencies [62] which characterize traditional ways of sample agitation. However, no studies have addressed this issue yet. No thorough comparison of mass transport enhancements by catalytic micromotors and by classic approaches was carried out. However, few papers do show that micromotors provide better results than classic stirring/agitation [63,64] (but without providing any explanation for the observed differences). Unlike classic ways to stir and mix samples, catalytic micromotors are also suitable to stir/mix very low volume samples (e.g., 10 µL of serum, saliva, or sweat placed on a glass microscope slide).
(ii). By enhancing the local concentration of optical/electrochemical probes. For example, SiO_2_-coated Ag nanowires are not only excellent probes for surface-enhanced Raman spectroscopy (SERS) but also show positive phototaxis, that is, they self-propel towards the light source via photocatalytic processes. The latter ability can be used to pre-concentrate the probes and improve the sensitivity and the detection limit of SERS-based detection [65]. Catalytic micromotors were also made using magnetic materials, such as Ni [17,19,46] or Fe_3_O_4_ [64]. In turn, these facilitated the magnetic pre-concentration of the catalytic micromotors onto the surface of the electrode for the electrochemical quantification of the analyte (which they have collected during self-propulsion in the investigated sample) [46,64].
(iii). By chemically transforming the targeted analyte. For example, Mg-based catalytic micromotors self-propel in aqueous solution while producing H_2_ and OH^-^ ions, and the latter species can facilitate the electrochemical detection of diphenyl phthalate by converting this, electrochemically inactive, compound into electrochemically active phenol [49]. A similar concept was also applied for the detection of paraoxon (a cholinesterase inhibitor) [66]. OH^-^ ions produced by catalytic micromotors facilitated also the detection of phenylenediamines by oxidizing these species to colored products [67].

**Figure 7:**
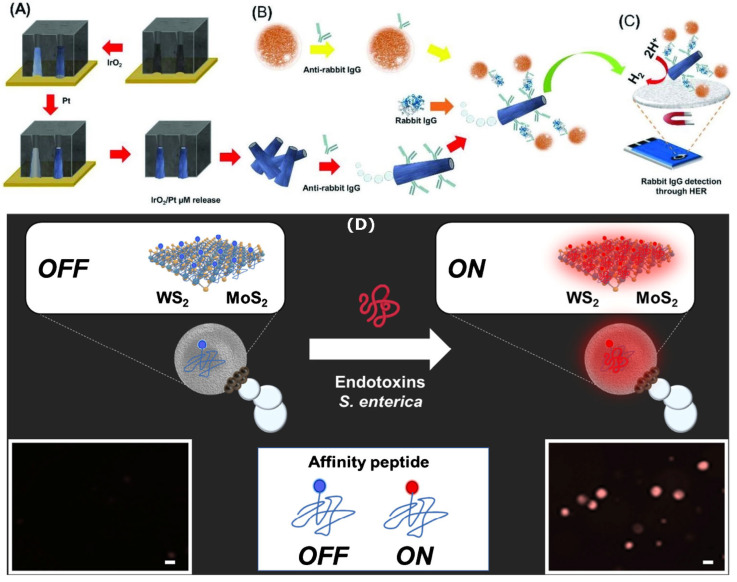
Schematic representation of the procedure to build IrO_2_ on Pt microtube catalytic micromotors (**A**), the formation of antibody–antigen–antibody complexes on the surface of the catalytic micromotors (**B**), the electrochemical quantification of the analyte concentration via the hydrogen evolution reaction (**C**), and the detection of bacteria endotoxin with catalytic micromotors which change fluorescence in the presence of the bacteria endotoxin (**D**); (Obs.: The targeted analyte is not involved in the catalytic process facilitating self-propulsion. However, self-propulsion is enhancing the probability of the catalytic micromotors to bind analyte molecules found in the sample.) Figures reproduced with permission from Ref. [68] (panels (**A**–**C**)) and Ref. [69] (panel (**D**)).

The structure of the catalytic micromotors increases in complexity when they are used to detect analytes not directly involved in the catalytic process propelling the catalytic micromotors. Such micromotors must carry not only the catalyst that facilitates the self-propulsion but also the recognition elements (which bind selectively the targeted analytes) and the optical/electrochemical labels (which facilitate quantifying the extent of target binding). To substantiate this idea, Figure 7 schematically shows the steps involved in the making and the using of catalytic micromotors for the detection of immunoglobulin G (IgG) (see also Ref. [68] for additional details). The micromotor depicted in Figure 7 has a Pt inner layer, which facilitates self-propulsion by the decomposition of H_2_O_2_, and an IrO_2_ outer layer with a dual role: to carry antibodies for the selective recognition of the analyte of interest and to act as catalyst for the hydrogen evolution reaction during the detection stage. The sensing concept also needs magnetic particle-labeled secondary antibodies (see Figure 7B), which will facilitate the concentration of the micromotors onto the surface of the electrode used in the detection stage (see Figure 7C). There are more than ten main steps involved in the making and the using of the catalytic micromotors. Such a high number of steps makes achieving reproducible measurements very difficult. Important to note: instead of using motion as the analytical signal, catalytic micromotor sensors of this category rely on either electrochemical signals (as shown in Figure 7C) or optical signals (as shown in Figure 7D).

Table 3 summarizes catalytic micromotors used for sensing species which are not directly involved in the catalytic processes of self-propulsion. In yet other words, the targeted analytes listed in Table 3 are neither fuel for the catalytic micromotors, nor inhibitors or activators of the catalytic processes facilitating self-propulsion.

If one compares Table 3 with Table 1 and Table 2, it becomes clear that sensing with catalytic micromotors is dominated by concepts which are not using the parameters of the motion of catalytic micromotors as the analytical signal.

There are several other points one can make based on the information summarized in Table 3. Instead of using the motion of catalytic micromotors as the analytical signal, the sensing concepts listed in Table 3 deliver either optical signals (76% of the micromotors) or electrochemical signals (24% of the micromotors). As such, these sensing concepts carry the problems characterizing the optical and electrochemical detection principles. For example, photobleaching remains a major problem of concepts based on fluorescence. Interferences and drifts (e.g., due to electrode fouling) can complicate biosensing concepts based on electrochemical detection. The catalytic micromotors get a new role in the concepts listed in Table 3 (as compared to the concepts listed in Table 1 and Table 2): they are used to enhance the mass transport in the investigated sample. Interestingly, the analytical signals obtained with mass transport enhanced by the self-propulsion of catalytic micromotors were found sometimes larger (e.g., in Refs. [78,81]), sometimes equal, and sometimes smaller (e.g., in Ref. [82]) than the analytical signals obtained with classic ways for stirring and mixing liquid samples (e.g., magnetic stirring). A thorough comparison of catalytic micromotors with classic tools for stirring and mixing liquid samples was not yet carried out. However, one must keep in mind that stirring and mixing liquid samples with catalytic micromotors requires no additional laboratory equipment and that there are very few tools to carry out stirring and mixing in samples of only few microliters volume. Enhancing the mass transport in the investigated sample was most often done with catalytic micromotors ejecting O_2_ bubbles by the decomposition of H_2_O_2_ (and only few times with micromotors which generate H_2_ bubbles via the reaction of Mg and H_2_O). Self-propulsion based on the ejection of O_2_ bubbles generated in H_2_O_2_, while more robust than phoretic self-propulsion, is still characterized by weaknesses (some of which were already mentioned in previous sections and will not be repeated here). O_2_ bubbles are only generated at high H_2_O_2_ concentrations (0.8–7.5%) and high H_2_O_2_ concentrations can oxidize sample components and cause problems both in electrochemical detection (as H_2_O_2_ is oxidized and reduced at relatively low applied potentials) and in optical detection (e.g., H_2_O_2_ concentrations higher than 7% were found to quench the CdTe quantum dots-based fluorescence of some catalytic micromotors [70]). In addition, the chemical composition of the investigated sample was also found to impact the speed of O_2_ bubble ejection-based catalytic micromotors. Some organic species (e.g., dimethyl sulfoxide) can quench radicals involved in the decomposition of H_2_O_2_ while others (e.g., thiols, furfural, and ethanol) can irreversibly adsorb onto the catalyst with detrimental effects on its ability to decompose H_2_O_2_ [86,87]. It is currently not clear (as it was not studied) how much the speed of the self-propulsion can decrease/increase (due to the combined effects of viscosity, organic species, temperature, etc.) without affecting the analytical signal in the sensing concepts listed in Table 3.

One can also note that selectivity tests are sketchy, in the best case, as the number of samples analyzed (after calibration with standard solutions) is very low (e.g., 2–3) in most of the available studies. The fact that catalytic micromotors were not tested with a larger number of complex (i.e., “real world”) samples is most likely due to the complexity of making the catalytic micromotors and using them for sensing purposes, as well to the (prohibitively large for most of the laboratories) time and costs demanded by such extensive validation studies.

## 6. Conclusions

Proof-of-principle studies of detection of analytes by using catalytic micromotors have been reported for a large number of very different type of analytes: e.g., metal ions, low molecular weight biomolecules, biomacromolecules, viruses, gases, etc. The analytes detected with catalytic micromotors are of interest in various fields, such as biomedicine, environment protection and remediation, and food safety. Few of these analytes were detected as they could also act as fuel for the catalytic micromotors (see Section 3). Other analytes were detected because they could modulate (e.g., inhibit or activate) the catalytic processes behind the self-propulsion of catalytic micromotors (without being the actual fuel of the catalytic micromotors; see Section 4). Most of the analytes were detected based on optical or electrochemical methods enhanced by catalytic micromotors. The ability of self-propelling catalytic micromotors to stir and mix the investigated sample was very important in this latter case (see Section 5). This ability is critical when the investigated sample is a tiny liquid droplet that cannot be stirred and mixed with classic laboratory equipment. Real-Time PCR [21,72], electrochemical biosensors [19,58,64,68,69,81,82], optical biosensors [19,58,64,69,75,81,82], enzyme-linked immunosorbent assays [22,64], and the limulus amoebocyte lysate test [69,81,82] are among the analytical tools compared to catalytic micromotor sensors. The analytical performances (most often the detection limit, sometimes the accuracy) of catalytic micromotor sensors were found sometimes better [64,68,75,81,82], sometimes similar [21,22,72,75,82], and sometimes worse [19,58,69,75] than those of the classic counterparts. The outcome of such a comparison obviously depends very much on the analytical tools selected as reference points. The shorter analysis times they facilitate and the extremely low volumes of sample they need are still undeniable advantages of the catalytic micromotor sensors.

However, as discussed in this review, there remain, in our opinion, a lot of aspects to be improved in what concerns the fabrication and use of catalytic micromotors as sensors. Most of the catalytic micromotors are produced by putting together several different materials via complicated, multistep procedures. We are clearly far from being able to mass produce highly reproducible, catalytic micromotors. Important properties (e.g., the speed of self-propulsion) of the currently produced catalytic micromotors show inconveniently large dispersions. Moreover, numerous details of the sample (e.g., viscosity, temperature, ion content, concentration of thiols, etc.), rather than just the concentration of the analyte of interest, seem to impact the behavior of catalytic micromotors, and thus the result of the “measurement”, in seemingly uncontrolled, and not well understood, ways. Most studies considered only a small number of “real-world” samples (e.g., 2–3) and thus the reliability of the sensing concepts based on catalytic micromotors remains poorly demonstrated; moreover, the proof-of-concepts studies are only seldom followed-up by thorough comparisons with reference analytical methods.

Accordingly, the conclusion that emerges from the available studies is that the qualitative (rather than quantitative) analysis of small samples in resource poor environments is the most promising niche area for the catalytic micromotors in analytical chemistry.

## Figures and Tables

**Table 1:** Examples of catalytic micromotors which were used to detect/quantify their fuel. (Used abbreviations: GOx = glucose oxidase, GluOx = glutamate oxidase, XOD = xanthine oxidase, DL = detection limit, and LR = linear range).

Targeted Analyte	Motor Structure; Self Propulsion Mechanism	Analytical Performances	Ref.
Glucose, glutamate and hypoxanthine	~2 µm long, half Pt and half poly(pyrrole) nanorods modified with GOx, GluOX or XOD; Self-diffusiophoresis;	DL: 0.05 mM for glucose, 0.03 mM for hypoxanthine, and 0.19 mM for glutamate; LR: up to 1 mM for glucose and glutamate and up to 0.1 mM for hypoxanthine; Selectivity proved in diluted horse serum and cell culture medium;	[6]
Hydrazine (vapors)	~1 µm diameter Au microsphere with one hemisphere covered with a 20 nm thick layer of Ir; Self-electrophoresis;	Vapors from a 2.5 mm diameter droplet containing 5–30% hydrazine induced self-propulsion of motors found in a second 2.5 mm diameter droplet (at 0.5–3 cm from the first);	[25]
Urea	~2 µm diameter hollow silica microcapsules modified with urease; Self-diffusiophoresis;	Self-propulsion was investigated at urea concentrations from 10 mM to 200 mM; Impact of the purity of urease on the speed of self-propulsion was discovered; Some stability problems were also noted;	[38]

**Table 2:** Examples of catalytic micromotors which were used to detect analytes which modulate the speed of self-propulsion without being fuel for the catalytic micromotors; (Used abbreviation: BSA = bovine serum albumin).

Targeted Analyte	Micromotor Structure; Self-Propulsion Mechanism	Analytical Performances	Ref.
*A. Catalytic micromotors used to detect inorganic compounds:*			
Ag^+^	Bimetallic nanorod (half Au and half Pt); Self-electrophoresis;	LR: from 0.5 µM to 100 μM; Interference from other ions was observed;	[39]
Hg^2+^	Halloysite clay nanotube (L ~700 nm, ϕ ~80–100 nm) partially covered with Pt; O_2_ bubble ejection;	DL: 3.24 ppb; Detected analyte concentrations from 0.25 ppb to 1000 ppb; The speed of the micromotors is also affected by other heavy metals;	[54]
Catalase inhibitors (Hg^2+^, Cu^2+^, NaN_3_, and aminotriazole)	PEDOT on Au microtube (L ~8 µm, ϕ ~2µm) with its inner surface modified with catalase; O_2_ bubble ejection;	Impact on speed observed for 50–200 μM Hg^2+^, 0.2–1 mM Cu^2+^, 2.5–25 μM NaN_3_, and 375–750 mM aminotriazole; The concept does not distinguish in between catalase inhibitors;	[55]
Pb^2+^	Cu on Pt microtube (ϕ =2 µm); O_2_ bubble ejection;	Detected concentrations: from 0.48 mM to 1.92 mM; Some selectivity was observed when tested with Cd^2+^; Interference from compounds able to adsorb onto Pt can be expected;	[56]
pH	Cylindrical gelatin cartridge (L ~15 µm, ϕ ~8 µm) with its inner surface decorated with 3 nm Pt nanoparticles; O_2_ bubble ejection;	Both the speed of self-propulsion and the distance traveled during 5 s were found proportional with pH; Sensitivity to pH from 0 to 14 was observed;	[57]
*B. Catalytic micromotors used to detect nucleic acids:*			
HIV-1 RNA	6 µm diameter polystyrene microbead modified with a patch of Au nanoparticles (~60 nm in diameter) and with Pt nanoparticles (~3 nm in diameter); O_2_ bubble ejection;	DL: 1000 virus particles/mL; Selectivity was proved by measurements in human serum; The approach needs RNA amplification;	[21]
DNA	PEDOT on Au microtube with its inner surface modified with capture DNA; O_2_ bubble ejection;	DL: 0.5 μM; The speed of the micromotors increased from 157 μm/s at 0.5 μM target DNA to 222 μm/s at 10 μM target DNA; Good selectivity observed with non-complementary DNA;	[51]
DNA	PEDOT on gold microtube (L ~13 µm, ϕ = 5 µm) modified with catalase via cyclic DNA hybridization; O_2_ bubble ejection;	Detected concentrations: from 10 nM to 1 μM; No selectivity was observed when tested with single-base mismatched DNA;	[52]
DNA	Multimetallic (Au/Ag/Ni/Au) shell with an opening of ~20µm and with its concave surface modified with catalase via DNA hybridization; O_2_ bubble ejection;	LR: from 25 nM to 750 nM and from 0.75 µM to 10 μM; Some interference from single-base and three-base mismatched DNA; 80% of initial speed maintained after 3 weeks;	[58]
DNA	Bimetallic nanorod (half Au and half Pt); Self-electrophoresis;	DL: 10 pM; Detected concentrations: from 10 pM to 100 nM; Some interference from two-base mismatched DNA; Relative standard deviation of 5.66%;	[59]
DNA	PEDOT on Au microtube (L ~13 µm, ϕ = 5 µm) modified with catalase via DNA conjugation; O_2_ bubble ejection;	Detected concentrations: from 0.5 µM to 10 μM; No selectivity was observed when tested with single-base mismatched DNA; Good functioning in spiked serum;	[60]
*C. Catalytic micromotors used to detect proteins:*			
Carcinoembryonic antigen	Poly(aniline) on Pt microtube (ϕ = 2 µm) with its outer surface modified first with 13 nm Au nanoparticles and then with antibodies; O_2_ bubble ejection;	Detected concentrations: from 1 ng/mL to 500 ng/mL; Good selectivity was observed when tested with alfa-fetoprotein and measurements in serum; Analysis time ~5 min; Relative standard deviation 7.8%;	[53]
*D. Catalytic micromotors used to detect pathogens:*			
Zika virus	Antibody-modified, ~5 nm diameter Pt nanoparticle that self-propels with H_2_O_2_; This motor attaches and subsequently propels 3 µm diameter, antibody-modified polystyrene microbeads only when the investigated sample contains the virus; O_2_ bubble ejection;	DL: 10 virus particles/µL; Selectivity was proved by measurements in urine, saliva, and serum samples;	[22]
*E. Catalytic micromotors used to detect small organic molecules:*			
Glutathione	20 µm diameter polystyrene microbead (covered with a 50 nm thick layer of Au and a layer of graphene oxide) carrying a patch of Pt nanoparticles; O_2_ bubble ejection;	DL: 0.90 μM; LR: up to 160 µM; Some interference from cysteine and BSA was observed; Good recoveries (>91%) in 100× diluted human serum;	[23]
Diethyl chlorophosphate	PEDOT on Au microtube (ϕ = 2 µm) with its inner surface modified with catalase; O_2_ bubble ejection;	Diethyl chlorophosphate vapors produced by a 0.1 mM solution were detected; Selectivity was observed when tested with non-volatile catalase inhibitors;	[61]

**Table 3:** Examples of micromotors which were used for sensing analytes which are not directly involved in the catalytic processes propelling the micromotors; (Used abbreviation: EDTA = ethylenediaminetetraacetic acid).

Targeted Analyte	Micromotor Structure; Self-Propulsion Mechanism	Analytical Performances	Ref.
*A. Catalytic micromotors used to detect inorganic compounds:*			
Cu^2+^ (heavy metals in generally)	Graphitic C_3_N_4_ microtube (L = 67 ± 14 µm, ϕ = 9.7 ± 1.5 µm); The micromotor could both sense and remove heavy metals; O_2_ bubble ejection;	Fluorescence detection; Cu^2+^ (from 1 ppm to 30 ppm) had the largest impact on the speed and the fluorescence of micromotors; 50% of 15 ppm Cu^2+^ was removed in 7 min;	[26]
Fe^3+^	~15 µm diameter microtube with a metal organic framework- based outer layer (functionalized with EDTA) and a layered double hydroxide- and MnO_2_-based inner layer (also functionalized with EDTA); O_2_ bubble ejection;	Fluorescence detection; DL: 0.15 µM; Measured concentrations: from 0.2 µM to 10 mM; LR: up to ~0.2 mM; Interference from other metal ions was observed; Adsorption capacity of 112 mg/g; Self-propulsion was turned on for the removal step while sensing was done in static conditions;	[27]
Hg^2+^	Layered microtube (L ~18 µm, ϕ ~2 µm) made of an inner layer of Pt and an outer layer of PEDOT modified with CdTe quantum dots; O_2_ bubble propulsion;	Fluorescence detection; 3 mg/L Hg^2+^ quenched the fluorescence in 12 s; The approach identifies as positive the samples with Hg^2+^ content > 0.3 mg/L; No effect of pH, ionic strength, Cu^2+^, Pb^2+^, or CH_3_Pb^+^;	[70]
Gaseous HCl and NH_3_	Hexagon-shaped, thiol-terminated polycaprolactone single crystal decorated with catalase; The micromotor also carried pH sensitive fluorescein isothiocyanate; O_2_ bubble ejection;	Fluorescence detection; DL: 50 ppm;	[71]
*B. Catalytic micromotors used to detect nucleic acids:*			
MicroRNA-155	~4.5 µm diameter, mesoporous microsphere partially covered with Pt nanoparticles and also modified with capture DNA probe; Self-diffusiophoresis;	Fluorescence detection; DL: 3.39 fM; LR: from 0.1 fM to 1 nM; Good selectivity observed when tested with single-base mismatched and three-base mismatched miRNA; Sensing functional in cell culture medium and cell lysates;	[72]
MicroRNA-21 and thrombin	MoS_2_ on Pt microtube (ϕ = 5 µm) modified with fluorescently labeled single-strand DNA (for microRNA-21) or aptamer (for thrombin); O_2_ bubble ejection;	Fluorescence detection; Detection of 0.2 µM miRNA-21 or 0.2 µM thrombin was demonstrated; Some interferences observed from single-base mismatch DNA strands and BSA;	[73]
MicroRNA-21	W_5_O_14_ nanowire (L ~10 µm, ϕ = 100 nm) modified with PEDOT, Pt and single stranded DNA; O_2_ bubble ejection;	Fluorescence detection; DL: 0.028 nM; LR: from 0.1 nM to 100 nM; Tests with single-base mismatch RNA strands highlighted some selectivity problems;	[74]
Methylated promoter region of Reprimo (RPRM) gene	Pt microtube (L ~12 µm, ϕ ~4 µm) modified with reduced graphene oxide and complementary single stranded DNA; O_2_ bubble ejection;	Fluorescence detection; DL: 1.3 µM; LR: from 1 µM to 10 µM; Some interference from non-complementary single stranded DNA was observed;	[75]
*C. Catalytic micromotors used to detect peptides and proteins:*			
C-reactive protein	Ni on Pt microtube (L ~10 µm, ϕ = 5 µm) modified with reduced graphene oxide and antibodies; O_2_ bubble ejection;	Electrochemical detection; DL: 0.8 µg/mL; LR: from 2 µg/mL to 100 µg/mL; Selectivity was proved in human plasma; Analysis time of 5 min;	[17]
C-reactive protein	Ni on Pt microtube (L ~10 µm, ϕ = 5 µm) modified with reduced graphene oxide and antibodies; O_2_ bubble ejection;	Electrochemical detection; DL: 0.4 µg/mL; LR: from 1 µg/mL to 100 µg/mL; Selectivity was proved in human serum and plasma; Analysis time of 8 min;	[18]
Procalcitonin	Ni on Pt microtube (L ~10–20 µm, ϕ = 5 µm) modified with polypyrrole and antibodies; O_2_ bubble ejection;	Fluorescence detection; DL: 0.07 ng/mL; LR: from 0.5 ng/mL to 150 ng/mL; Selectivity was proved in human serum; Analysis time of 30 min;	[19]
Procalcitonin	Dynabeads modified with anti-procalcitonin antibodies (which facilitate a competitive immunoassay with catalase labeled procalcitonin); O_2_ bubble ejection;	Colorimetric detection; DL: 2 ng/mL; LR: from 1 ng/mL to 20 ng/mL; Selectivity was proved in human whole blood; Analysis time of 13 min;	[20]
IgG	~500 nm Fe_3_O_4_ core and SiO_2_ shell nanoparticle modified with Pt and also with anti-IgG antibodies; Self-diffusiophoresis;	Electrochemical detection; DL: 3.14 pg/mL; LR: from 10 pg/mL to 100 ng/mL; Interference from IgA was observed; ~20% of the signal lost after 15 days of storage at 4 °C;	[64]
Rabbit IgG	IrO_2_ on Pt microtube (L ~10 µm, ϕ ~2.5 µm) modified with anti-rabbit IgG; O_2_ bubble ejection;	Electrochemical detection; DL: 0.94 pg/mL; LR: from 0.05 ng/mL to 500 ng/mL; Poor selectivity when tested with hemoglobin; Relative standard deviation of ~11%;	[68]
Phycocyanin	Microtube (L ~18 µm, ϕ ~2 µm) made of layers of Pt, Ni, and PEDOT imprinted with analyte molecules; O_2_ bubble ejection;	Fluorescence detection; Measured concentrations: 0.5, 0.75, and 1 mg/mL; BSA and seawater salts did not interfere;	[76]
Ricin B	Reduced graphene oxide on Pt microtube (L ~10 µm, ϕ ~5 µm) modified with aptamer; O_2_ bubble ejection;	Fluorescence detection; LR: from 100 pg/mL to 10 μg/mL; Tests with BSA and saporin indicated good selectivity;	[77]
*D. Catalytic micromotors used to detect pathogen toxins:*			
Cholera toxin B	Graphdiyne on Pt microtube (L = 10–20 µm, ϕ ~5 µm) modified with rhodamine labeled affinity peptide; O_2_ bubble ejection;	Fluorescence detection; DL: 1.6 ng/mL; LR: from 4.5 ng/mL to 5000 ng/mL; Good selectivity when tested with *Escherichia coli* toxin and BSA; Good recoveries when tested with serum samples;	[24]
Cholera toxin B	20 µm diameter polystyrene bead covered with graphdiyne oxide and carrying a patch of Pt and Fe_2_O_3_ nanoparticles and also recognition peptides; O_2_ bubble ejection;	Fluorescence detection; DL: 0.002 µg/mL; LR: from 0.008 µg/mL to 10 µg/mL; Selectivity in complex samples (e.g., human serum) was observed;	[78]
Fumonosin B1	Ni on Pt microtube (L ~10 µm, ϕ = 5 µm) modified with reduced graphene oxide; Selectivity assured with labeled aptamer; O_2_ bubble ejection;	Fluorescence detection; DL: 0.7 ng/mL; LR: from 0.005 µg/mL to 1 µg/mL; Measurements in beer were made; Ochratoxin A was also detected using the same method;	[63] (see also [79])
*Salmonella enterica* endotoxin	~10 µm diameter polycaprolactone microspheres loaded with transition metal dichalcogenides carrying fluorescent recognition peptides, 50 nm Pt nanoparticles, and 20 nm Fe_2_O_3_ nanoparticles; O_2_ bubble ejection;	Fluorescence detection; DL: 1.2 μg/mL; LR: from 4 μg/mL to 333 μg/mL; Good selectivity when tested with endotoxins from other bacteria; Recoveries > 93%; Analysis time ~5 min;	[69]
*Escherichia coli* O111:B4 lipopolysaccharide	~20 µm diameter polycaprolactone microspheres loaded with graphene quantum dots carrying phenylboronic acid, Pt nanoparticles, and Fe_2_O_3_ nanoparticles; O_2_ bubble ejection;	Fluorescence detection; Measured concentrations: from 10 mM to 1 M; No interference from 2 M glucose, fructose, or galactose; Detecting high concentrations of liposaccharide (e.g., 2 M) in urine and serum samples was possible;	[80]
*Escherichia coli* O111:B4 lipopolysaccharide	~25 µm diameter polycaprolactone microspheres loaded with layered WS_2_ carrying fluorescent recognition peptides, Pt nanoparticles, and Fe_2_O_3_ nanoparticles; O_2_ bubble ejection;	Fluorescence detection; DL: 120 pM; LR: from 4 ng/mL to 1 mg/mL; Selectivity was proved with non-target lipopolysaccharide endotoxins and measurements in human serum; Analysis time of 5 min;	[81]
*Salmonella enterica* lipopolysaccharide	~20 µm diameter polycaprolactone microbead containing 100 nm Pt nanoparticles and graphene quantum dots carrying receptor molecules; O_2_ bubble ejection;	Fluorescence detection; DL: 0.07 ng/mL; LR: up to 1 ng/mL; Analysis time of 15 min; Measurements in milk, mayo, egg yolk, and egg white were made;	[82]
*E. Catalytic micromotors used to detect small organic molecules:*			
L-tryptophan	Ni on Pt microtube (L = 10–20 µm, ϕ = 2–3 µm) modified with CdS quantum dots and with β-cyclodextrin; O_2_ bubble ejection;	Fluorescence and electrochemical detection; Some preference for L-tryptophan is demonstrated; No other analytical performances were detailed;	[46]
2,4,6-Trinitrophenol	~30–40 µm diameter polycaprolactone microspheres loaded with fluorescent covalent-organic-frameworks, MnO_2_ microurchins, and Fe_3_O_4_ nanoparticles; O_2_ bubble ejection;	Fluorescence detection; 2,4,6-trinitrophenol turns off the fluorescence of the micromotors; Detection of 1 ppm of 2,4,6-trinitrophenol was demonstrated;	[47]
Diphenyl phthalate	~20 µm diameter Mg microparticles partially covered with Au; OH^-^ ions, which degrade diphenyl phthalate to phenol, are also produced during self-propulsion; H_2_ bubble ejection;	Electrochemical detection; DL: 0.039 mM; LR: from 0.12 mM to 1 mM; Recovery > 97 ± 8%; Analysis time ~5 min; Analysis of tap water, whiskey, milk, and human serum samples was successfully carried out;	[49]
Crystal violet (as model analyte)	SiO_2_-coated Ag nanowire (~150 nm in diameter, L ~15 µm) with a spherical AgCl head; Self-electrophoresis;	SERS detection; Measured concentration: 0.1 mM; Positive phototaxis of the micromotors led to an increase in the analytical signal (e.g., 6.2×);	[65]
Methyl paraoxon	~40 µm diameter Mg microbead partially covered with a 80 nm thick layer of Ni and a 10 nm thick layer of Au; H_2_ bubble ejection;	Electrochemical detection; LR: from ~5 mM to 20 mM; Relative standard deviation < 5%;	[66]
Ortho-phenylenediamine	Carbon nanotubes and 50 nm Fe_2_O_3_ nanoparticles on MnO_2_ microtube (L ~12 µm, ϕ ~2 µm); O_2_ bubble ejection;	Colorimetric detection; DL: 5 µM; LR: from 16.7 µM to 500 μM; Analysis time ~15 min; Interference from 20 µM Cu^2+^ and 20 µM Fe^3+^ was observed; Similar results were obtained for para-phenylenediamine;	[67]
Diethyl chlorophosphate (nerve agent simulant)	Silica particle covered with fluoresceinamine and then partially covered with Pt; O_2_ bubble ejection;	Fluorescence detection; 10 mM diethyl chlorophosphate is detected in 1 min while 10 µM diethyl chlorophosphate is detected in about 3 min; Selectivity was proved with ethanol, toluene, acetone, and isopropanol;	[83]
Cortisol	Microtube (L ~10 µm, ϕ = 5 µm) made of an outer layer of PEDOT, a middle layer of Ni and an inner layer of Pt; The outer layer was further modified with Au and anti-cortisol antibodies; O_2_ bubble ejection;	Colorimetric detection; Lowest detected concentration 0.1 μg/mL; Analysis time 2 min;	[84]
Glucose	~30 µm diameter Mg microbead partially covered with Pt; H_2_ bubble ejection;	Electrochemical detection; DL: 33.2 µM; LR: from 1 mM to 15 mM; Measurements in diluted human serum were made;	[85]

## Data Availability

Not applicable.
